# The steroidal lactone withaferin A impedes T-cell motility by inhibiting the kinase ZAP70 and subsequent kinome signaling

**DOI:** 10.1016/j.jbc.2021.101377

**Published:** 2021-11-03

**Authors:** Mobashar Hussain Urf Turabe Fazil, Chandra Sekhar Chirumamilla, Claudina Perez-Novo, Brandon Han Siang Wong, Sunil Kumar, Siu Kwan Sze, Wim Vanden Berghe, Navin Kumar Verma

**Affiliations:** 1 Lee Kong Chian School of Medicine, Nanyang Technological University Singapore, Clinical Sciences Building, Singapore; 2 Laboratory of Protein Chemistry, Proteomics and Epigenetic Signaling (PPES) and Integrated Personalized and Precision Oncology Network (IPPON), Department of Biomedical Sciences, University of Antwerp, Wilrijk, Belgium; 3 NTU Institute for Health Technologies (HealthTech NTU), Interdisciplinary Graduate Programme, Nanyang Technological University Singapore, Singapore; 4 Indian Council of Agricultural Research-National Bureau of Agriculturally Important Microorganisms, Kushmaur, Mau, Uttar Pradesh, India; 5 School of Biological Sciences, Nanyang Technological University Singapore, Singapore

**Keywords:** T-lymphocytes, migration, withaferin A, inflammation, kinome, DTT, dithiothreitol, ICAM-1, intercellular adhesion molecule-1, IKKβ, I-kappa-B-kinase β, LFA-1, lymphocyte function-associated antigen-1, MAP, mitogen-activated protein, NFκB, nuclear factor kappa B, PBS, phosphate buffered saline, PKC, protein kinase C, PTK, protein tyrosine kinase, rICAM-1, recombinant ICAM-1, SDF-1α, stromal cell-derived factor 1 α, STK, serine/threonine kinase, TCR, T-cell receptor, TNF-α, tumor necrosis factor-α, WFA, Withaferin A, ZAP70, zeta-chain-associated protein kinase 70

## Abstract

The steroidal lactone withaferin A (WFA) is a dietary phytochemical, derived from *Withania somnifera*. It exhibits a wide range of biological properties, including immunomodulatory, anti-inflammatory, antistress, and anticancer activities. Here we investigated the effect of WFA on T-cell motility, which is crucial for adaptive immune responses as well as autoimmune reactions. We found that WFA dose-dependently (within the concentration range of 0.3–1.25 μM) inhibited the ability of human T-cells to migrate *via* cross-linking of the lymphocyte function-associated antigen-1 (LFA-1) integrin with its ligand, intercellular adhesion molecule 1 (ICAM-1). Coimmunoprecipitation of WFA interacting proteins and subsequent tandem mass spectrometry identified a WFA-interactome consisting of 273 proteins in motile T-cells. In particular, our data revealed significant enrichment of the zeta-chain-associated protein kinase 70 (ZAP70) and cytoskeletal actin protein interaction networks upon stimulation. Phospho-peptide mapping and kinome analysis substantiated kinase signaling downstream of ZAP70 as a key WFA target, which was further confirmed by bait-pulldown and Western immunoblotting assays. The WFA-ZAP70 interaction was disrupted by a disulfide reducing agent dithiothreitol, suggesting an involvement of cysteine covalent binding interface. *In silico* docking predicted WFA binding to ZAP70 at cystine 560 and 564 residues. These findings provide a mechanistic insight whereby WFA binds to and inhibits the ZAP70 kinase and impedes T-cell motility. We therefore conclude that WFA may be exploited to pharmacologically control host immune responses and potentially prevent autoimmune-mediated pathologies.

T-cell motility is an intrinsic attribute that ensures immuno-surveillance as well as mounting of an adaptive immune response (1). At the local site of inflammation, T-cells migrate to facilitate homeostasis as well as resolve tissue damage by a coordinated array of regulated signal transduction and cytoskeletal remodelling (2).

The process of T-cell migration is primarily mediated by the interaction between the T-cell αLβ2 integrin lymphocyte function-associated antigen-1 (LFA-1) and the ligand intercellular adhesion molecule-1 (ICAM-1), expressed on the endothelium (3, 4, 5). This receptor/ligand engagement also plays a critical role in regulating T-cell extravasation into the lymph node and the direction of T-cell migration in the blood vessel (6). Following LFA-1/ICAM-1 engagement and firm adhesion, T-cells crawl along the endothelium exploring a site to migrate across the endothelial lining into the tissue (7). Notably, the passage through endothelium, which starts with ICAM-1-dependent intraluminal crawling followed by crossing of the endothelial monolayer, also occurs in the absence of ICAM-1. In the next step, T-cells colocalizing with the basement membrane are retained in an ICAM-1-independent manner before entering the lymph node parenchyma (8). The motility of lymphocytes within the parenchyma of a lymphoid tissue is modestly reduced in the absence of ICAM-1, which is acknowledged as ICAM-1-independent migration. The interaction between LFA-1 and ICAM-1 activates numerous kinases triggering downstream signaling cascades that facilitate cytoskeletal remodelling and T-cell motility (9, 10, 11).

Withaferin A (WFA) is an archetype withanolide, discovered from the root extract of *Withania somnifera*. Growing evidence has shown that WFA possesses immunomodulatory, antimicrobial, and anticancer activities (12, 13). Little is known about the effect of this compound on T-lymphocyte migration.

Previous studies using various cell types have shown a broad range of WFA targets, including kinases such as I-kappa-B-kinase β (IKKβ) and p38 mitogen-activated protein (MAP) kinase (14, 15, 16, 17). However, an integrated kinome-wide response and the molecular basis for such biological activities of WFA on T-cell motility are unclear.

In the current study, we determined the effects of WFA on the migratory behavior of human T-lymphocytes. We performed a phospho-peptidome analysis using phospho-peptide substrate microarrays and identified kinase targets of WFA in LFA-1-stimulated T-cells. Proteomics, *in silico* analysis, and molecular assays confirmed WFA-interaction partners in motile T-cells. We demonstrate that WFA inhibits T-cell motility by a mechanism involving an interplay between signaling cascades downstream of the zeta-chain-associated protein kinase 70 (ZAP70) and the actin cytoskeleton.

## Results

### WFA inhibits LFA-1/ICAM-1-stimulated T-cell motility without impacting cell adhesion on ICAM-1

**Figure 1:**
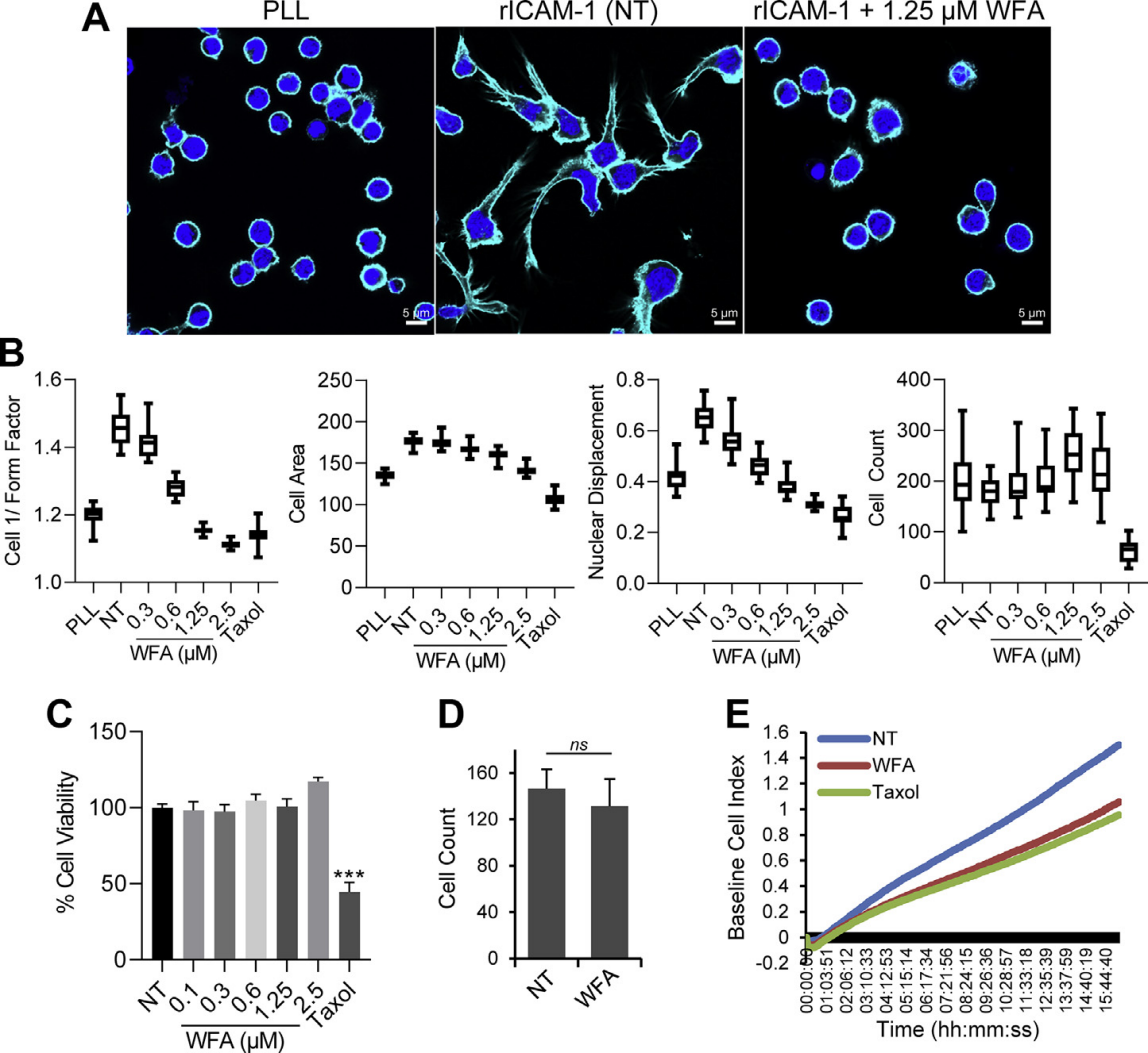
**WFA inhibits T-cell migration.** Human primary T-cells (2 × 10^4^ cells/per well in 96-well plates in triplicates) were pretreated with increasing concentrations of WFA (0.1–2.5 μM) for 3 h or left untreated (*NT*, treated with 0.001% DMSO as a control). Cells were allowed to migrate on immobilized rICAM-1. Resting cells were placed on poly-L lysine (*PLL*)-coated plate. *A*, cells were fixed after 30 min of migration, stained with Hoechst (*blue*) to visualize nuclei and Alexa Fluor phalloidin 647 (*cyan*) to visualize cell periphery/actin cytoskeleton and imaged by an automated microscopy. Scale bar = 5 μM. *B*, box plots showing interquartile range with median values recorded in 16 random fields from high content analysis—evaluated for cell 1/form factor, cell area, nuclear displacement, and cell count/field. *C*, viability of cells was determined using an MTS-based assay and absorbance readings normalized against *NT* is plotted as % cell viability (mean ± SD); ∗∗∗*p* < 0.0001. *D*, primary T-cells without or with pretreatment with 1.25 μM WFA were analyzed for adhesion onto rICAM-1-coated surface under continuous shear flow. Number of cells adhering on rICAM-1 in 50 s was double-blind counted and presented; *ns*, nonsignificant. *E*, real-time chemotaxis determination by impedance-based measurements of WFA-treated (1.25 μM) human primary T-cells toward the chemokine SDF-1α. NT migrating cells and cells treated with taxol were used as controls. Baseline was drawn automatically for wells without SDF-1α. Data is representative of at least three independent experiments using T-cells from at least three different donors.

Immediately after engagement of the LFA-1 receptor, T-cells polarize and undergo dynamic cytoskeletal remodeling exhibiting a tadpole-like migratory phenotype. To determine the effect of WFA on T-cell motility, we pretreated human primary T-cells with increasing concentrations of WFA (0.3–2.5 μM) for 3 h and then seeded into the wells of 96-well plates that were fully coated with recombinant ICAM-1 (rICAM-1). T-cells adhering to the immobilized rICAM-1 acquired a distinct polarized and elongated motile morphology. However, while WFA-treated cells adhered to the rICAM-1-coated surface, they displayed loss of migratory morphologies appearing round-shaped (Fig. 1*A*). Multiparametric quantitation of migratory phenotypes employing high content analysis showed that WFA dose-dependently decreased cell 1/form factor (a measure of cell polarity), cell area, and nuclear displacement; 1.25 μM WFA completely inhibited T-cell migration without impacting the number of cells adhered to immobilized rICAM-1 (Fig. 1*B*) and cell viability (Figs. 1*C* and S1). Primary T-cells exposed to WFA (1.25 μM for 3 h) were able to adhere on the rICAM-1-coated surface under continuous flow (0.5 dyne/cm^2^) in the same way as untreated cells (Fig. 1*D*, Movies S1 and S2). Therefore, this concentration of WFA (1.25 μM) was chosen for further analysis.

We next used a transwell migration assay to assess the effect of WFA on T-cell chemotaxis toward the chemokine stromal cell-derived factor 1 α (SDF-1α). Primary T-cells pretreated with 1.25 μM WFA showed significantly reduced chemotaxis in comparison to the control, as quantified by impedance-based measurements in real time (Fig. 1*E*). Since WFA did not interfere with the initial T-cell adhesion on ICAM-1 but inhibited LFA-1/ICAM-1-induced T-cell motility and chemotaxis, it could imply that WFA affects downstream intracellular signaling cascades.

### WFA-interactome in LFA-1/ICAM-1-stimulated motile T-cells

**Figure 2:**
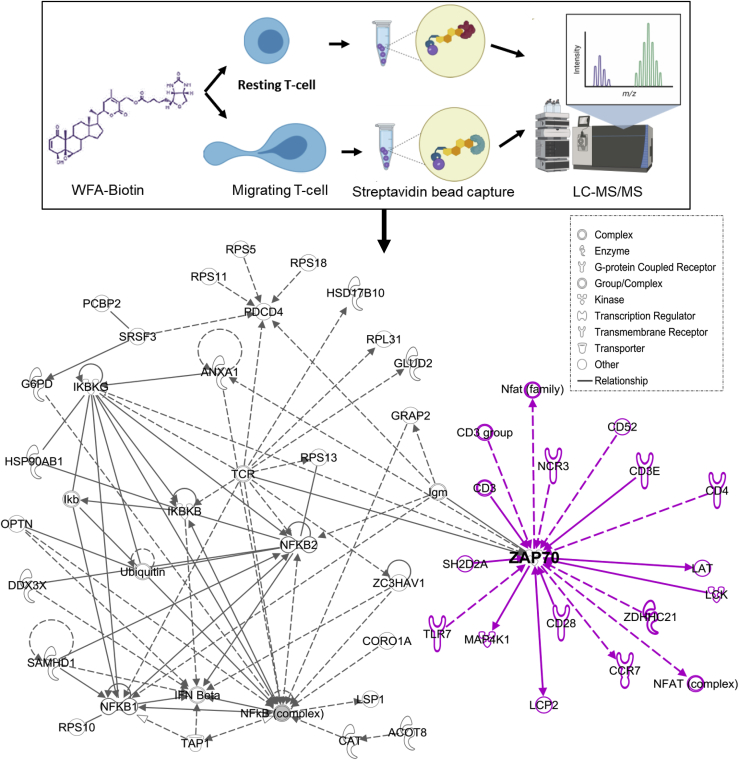
**Identification of WFA interactome in migrating T-cells.** Schematic representation of workflow employed in mass-spectrometry-based proteomics analysis of WFA interactome in human T-cells. An image output from IPA indicating statistically probable interactions of WFA in LFA-1-stimulated T-cells is shown. A maximum of 20 direct/indirect interactions upstream/downstream of ZAP70 were added by the Ingenuity Knowledge Base, shown in *purple*. The *continuous solid lines* and *broken lines* indicate direct and indirect interactions, respectively. The type of network, shapes in IPA are provided in the legend.

To identify WFA target intracellular molecules (WFA-interactome) in LFA-1/ICAM-1-stimulated motile T-cells, we pulled down cellular proteins using WFA-Biotin and streptavidin beads and performed mass spectrometry analysis. A schematic of the proteomics workflow is depicted in Figure 2. A total of 273 unique proteins were identified using this method (Table S1). In particular, we identified several kinases in the T-cell WFA-interactome, including ZAP70, IKKβ, STK10, and pyruvate kinase, in addition to mitochondrial enzymes (methylcrotonoyl-coA carboxylase 1, citrate synthase, glutamate dehydrogenase), cytoskeletal proteins (filamin, myosin, vimentin, keratin, actin, tubulin), glucose 6 phosphate dehydrogenase, Ras-GTPase activating protein, and fatty acid synthase (Table S1). Ingenuity Pathway Analysis (IPA) revealed a central node with ZAP70, interconnected *via* the T-cell receptor (TCR) and the nuclear factor kappa B (NFκB) pathways in WFA-treated LFA-1/ICAM-1-stimulated T-cells (Fig. 2).

### WFA targets cellular kinases involved in LFA-1/ICAM-1-stimulated T-cell motility

**Figure 3:**
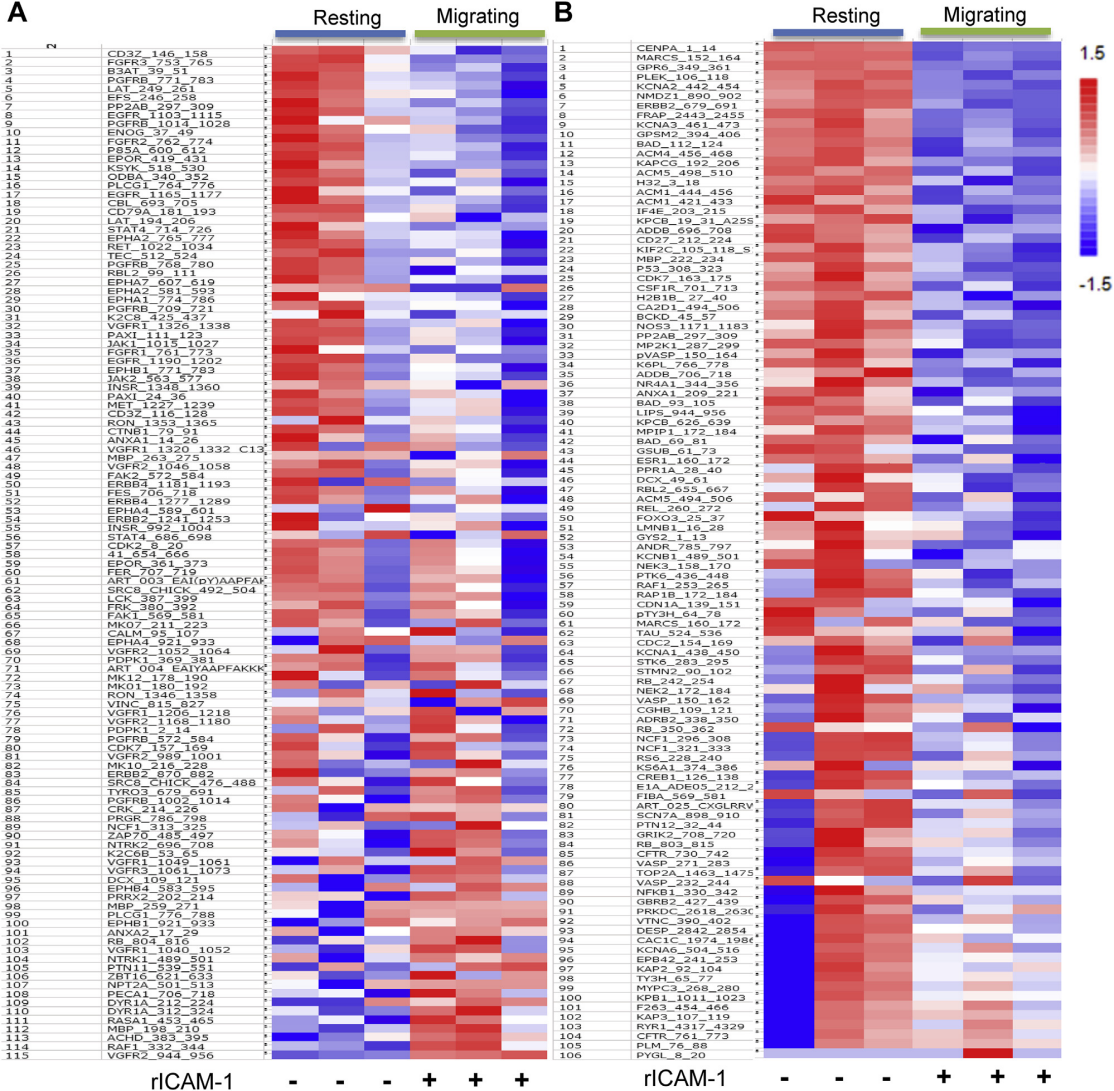
**Heat maps showing phosphopeptide intensities.** Human primary T-cells were seeded to migrate on a rICAM-1-coated plate for 30 min and lysed. Cellular lysates were processed for PamChip Peptide array analysis. Heat maps were plotted with calculated signal values based on intensity calculations of phosphorylated peptides on PTK array (*A*) and STK array (*B*). Peptide Uniprot ID's along with the range of amino acid sequences employed in the array is indicated on the *left side* of each panel.

**Figure 4:**
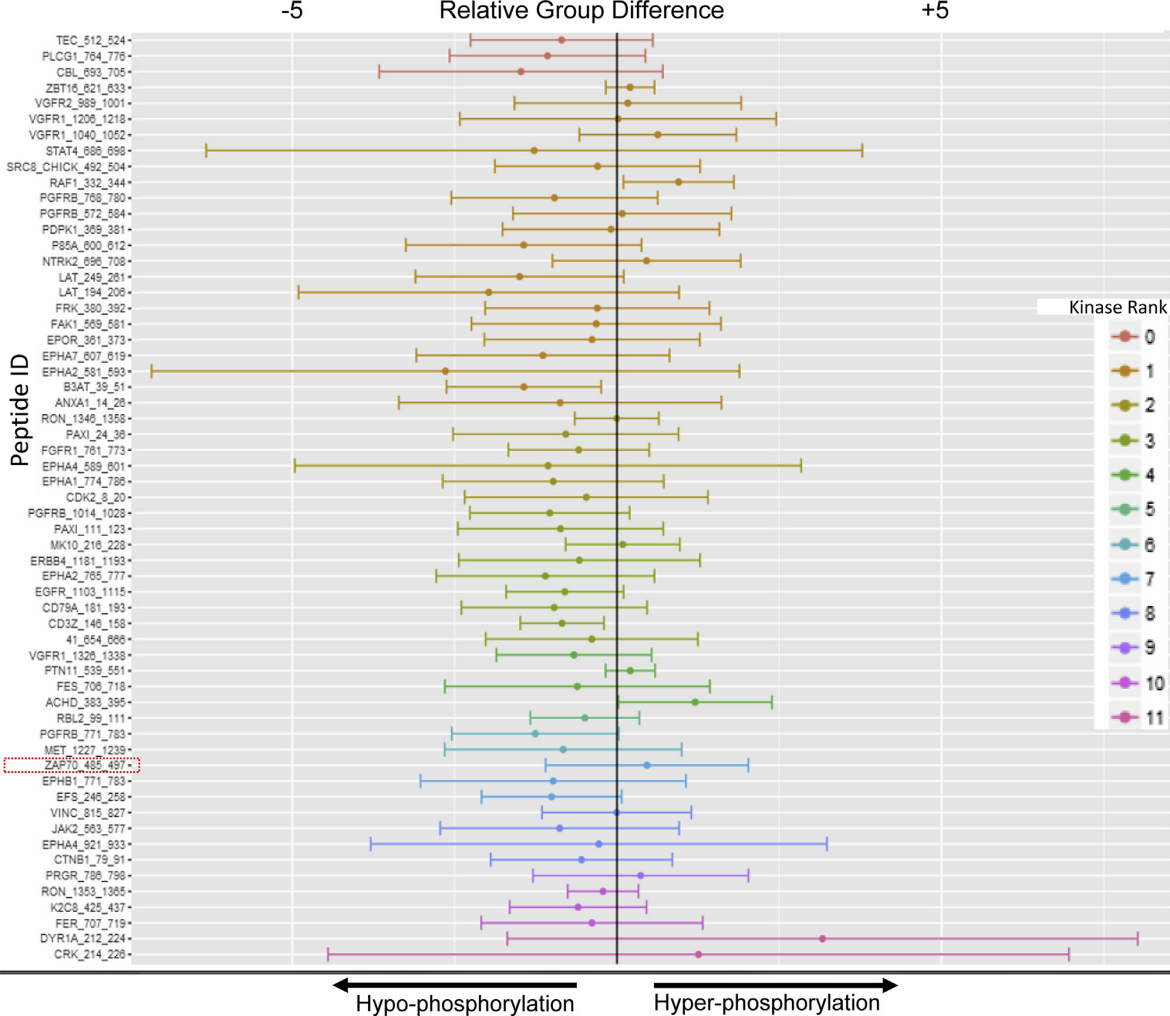
**ZAP70-phosphopeptidome analysis in migrating T-cells.** A peptide plot with *x*-axis showing the differences in peptide phosphorylation between LFA-1-stimulated migrating T-cells in comparison to resting controls. The Zap70_485_497 peptide specific phosphorylation shows hyperphosphorylation in migrating T-cells. Upstream kinases analysis of ZAP70-linked-phosphopeptidome indicates a hypo-phosphorylation of most associated kinases after 30 min of LFA-1/ICAM-1 stimulation compared with control. Peptide ranking is based on confidence limit of experimental evidence (*in vitro* + *in vivo*) and the incidence in all major protein databases—HPRD, PhosphoELM, PhosphositePLUS, Reactome, and UNIPROT.

A preliminary comparison of unstimulated resting and LFA-1/ICAM-1-stimulated T-cells indicated a significant change in the overall kinome profile of migrating T-cells (Fig. 3). Average phosphopeptide intensity calculations of the peptides representing KCNA2/3, FRAP, TAU, and RB1 within the serine/threonine kinase (STK) group and PGFRB and CD3ζ within the protein tyrosine kinase (PTK) family were repressed in motile T-cells (Fig. 3). Reciprocally, increased intensities were observed for DYRK1A, CRK, EPHB1, MAPK10, glycogen phosphorylase, phospholemman, and CFTR-specific phosphopeptides in LFA-1/ICAM-1-stimulated migrating T-cells as compared with unstimulated resting T-cells. Although ZAP70 peptide phosphorylation on the array was increased in LFA-1/ICAM-1-stimulated motile T-cells, upstream kinase analysis of the total phosphopeptidome linked to ZAP70 within the array showed an overall diminution in related-kinases activity in motile T-cells (Fig. 4). This suggests an ongoing dynamic kinases/phosphatases interplay with function of time in LFA-1/ICAM-1-stimulated migrating T-cells. This may also be due to loss of information regarding activity in upstream kinase analysis, as multiple peptide spots may correspond to unique sites on a specific protein in certain cases.

**Figure 5:**
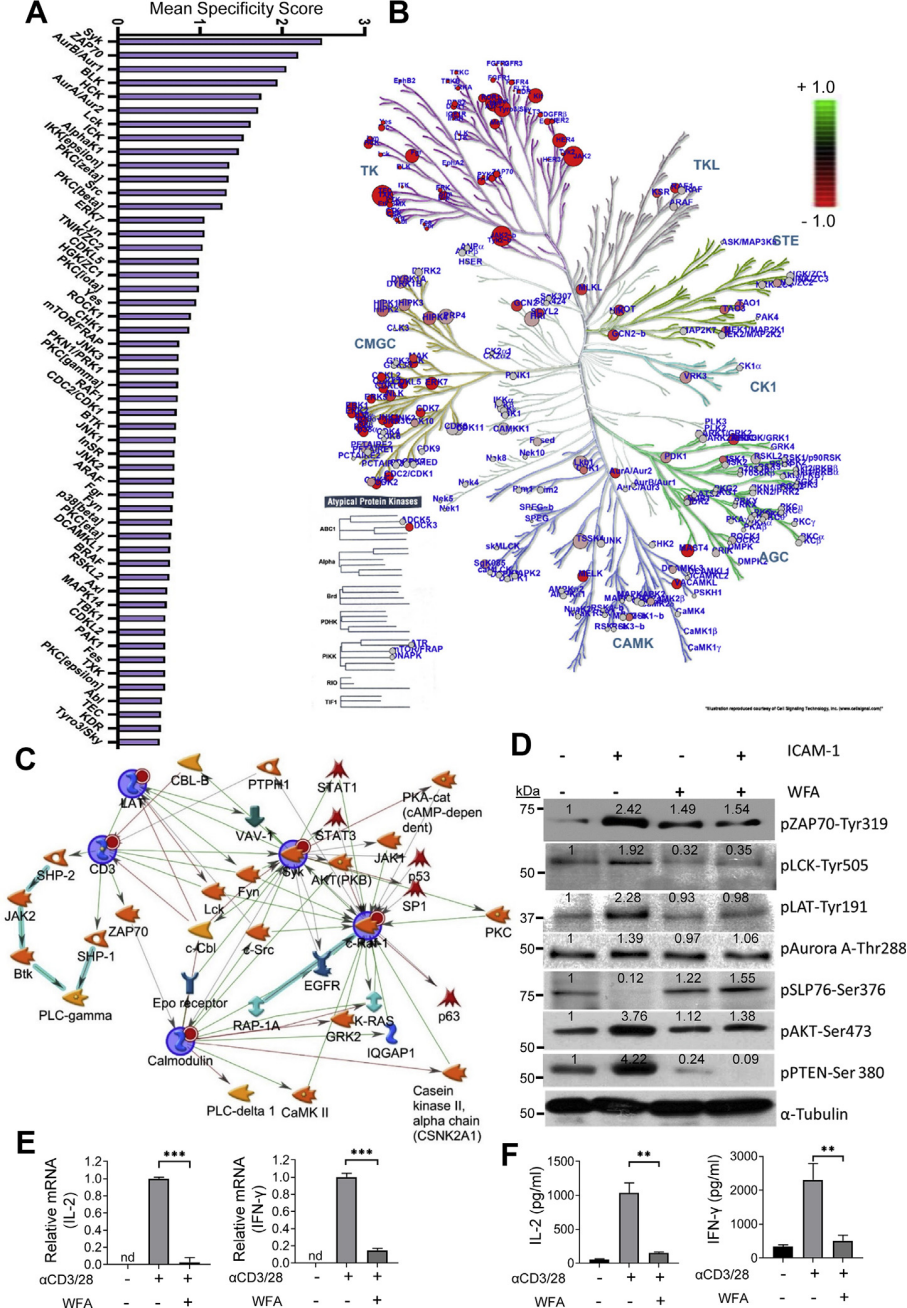
**Kinase activity profiling of WFA-treated human T-lymphocytes.***A*, mean specificity score calculations signifying the specificity in terms of set of peptides used for the corresponding kinase in WFA-treated LFA-1/ICAM-stimulated migrating T-cells. A higher score indicates higher specificity and less randomness in peptide assignment for the matching kinase. *B*, kinase dendrogram complementing the kinome identified in WFA treated LFA-1/ICAM-1-stimulated T-cells. Kinases from nearly all groups of the human kinome were detected: CK1, casein kinases; CMGC, CDK/MAPK/GSK3/CLK-family kinases; RCG, receptor guanylate cyclases; STE, sterile homologue kinases; TK, tyrosine kinases; TKL, tyrosine kinase-like kinases; atypical protein kinases. The size of the *circle dot* indicates the specificity score of the corresponding kinases compared with untreated controls, whereas the color indicates relative activity (*red* indicates less active and green hyperactive relative to untreated migrating T-cells control samples). Data represent at least three experiments performed by using T-cells isolated from three different donors. *C*, network analysis of kinases identified in WFA-treated T-cell kinome. Image generated by GeneGo MetaCore (Clarivate Analytics). *D*, western immunoblot analysis of pZAP70-Tyr319, pLCK-Tyr505, pLAT-Tyr191, pAurora A-Thr288, pSLP76-Ser376, pAKT-Ser473, and pPTEN-Ser380 in 1.25 μM WFA-treated T-cells stimulated without or with rICAM-1. The blot was reprobed with anti-α-tubulin as a loading control. Primary T-cells were reactivated *via* anti-CD3/28 TCR in the absence or presence of 1.25 μM WFA. Cellular levels of IL-2 and IFN-γ mRNA were quantified by RT-qPCR (*E*) and the secreted levels of IL-2 and IFN-γ were determined by ELISA (*F*). Data are mean ± SEM from primary T-cells obtained from four different donors performed in triplicates; ∗∗*p* < 0.01; ∗∗∗*p* < 0.001; *nd*, not detected.

We then performed PamChip peptide microarray to determine the impact of WFA on downstream cellular kinases in LFA-1/ICAM-1-stimulated motile T-cells. Kinase specificity scores of WFA-treated T-cells upon stimulation *via* LFA-1/ICAM-1 engagement showed that SYK, ZAP70, AURORA A/B, LCK, and mTOR kinases were among the top kinases constrained by WFA (Fig. 5*A*). In addition, kinase statistical calculations (a measure of kinase activity) predicted TXK, JAK2, FAK, MER, ALK, and ZAP70 PTK's and PKCε, CAMK4, MAPKAPK2, ERK2, and AKT1 STKs were inhibited by WFA in LFA-1/ICAM-1-stimulated T-cells (Fig. S2). An integrated view of kinase activity modulations by WFA, obtained by upstream kinase analysis, is summarized in the kinase dendrogram (Fig. 5*B*). GeneGo MetaCore analysis of the kinome datasets designated a role for ZAP70, LCK, LAT, AKT, and STAT3 in the inhibition of LFA-1/ICAM-1-stimulated T-cell migration by WFA (Fig. 5*C*). Further validation using Western immunoblot analysis confirmed WFA-mediated inhibition of LFA-1/ICAM-1-induced phosphorylation including ZAP70 (pZAP70-Tyr319), LAT (pLAT-Tyr191), LCK (pLCK-Tyr505) AKT (pAKT-Ser473), PTEN (pPTEN-Ser380), and AURORA kinase A (pAurora A-Thr288) in migrating T-cells (Fig. 5*D*). At the same time, WFA treatment increased the inactivating phosphorylation of the adaptor protein SLP76 (pSLP76-Ser376) in LFA-1/ICAM-1-stimulated motile T-cells (Fig. 5*D*).

Since the identified WFA target PTKs and STKs are important for TCR signaling, we tested potential functional impact of WFA on TCR-induced T-cell activation. Primary T-cells were reactivated *via* CD3/28 TCR in the presence or absence of 1.25 μM WFA. RT-qPCR and ELISA assays showed that WFA significantly inhibited TCR-induced production of IL-2 and IFN-γ by activated primary T-cells (Fig. 5, *E* and *F*).

### Molecular modeling predicts WFA binding to ZAP70 at cysteine 560 and 564 residues

**Figure 6:**
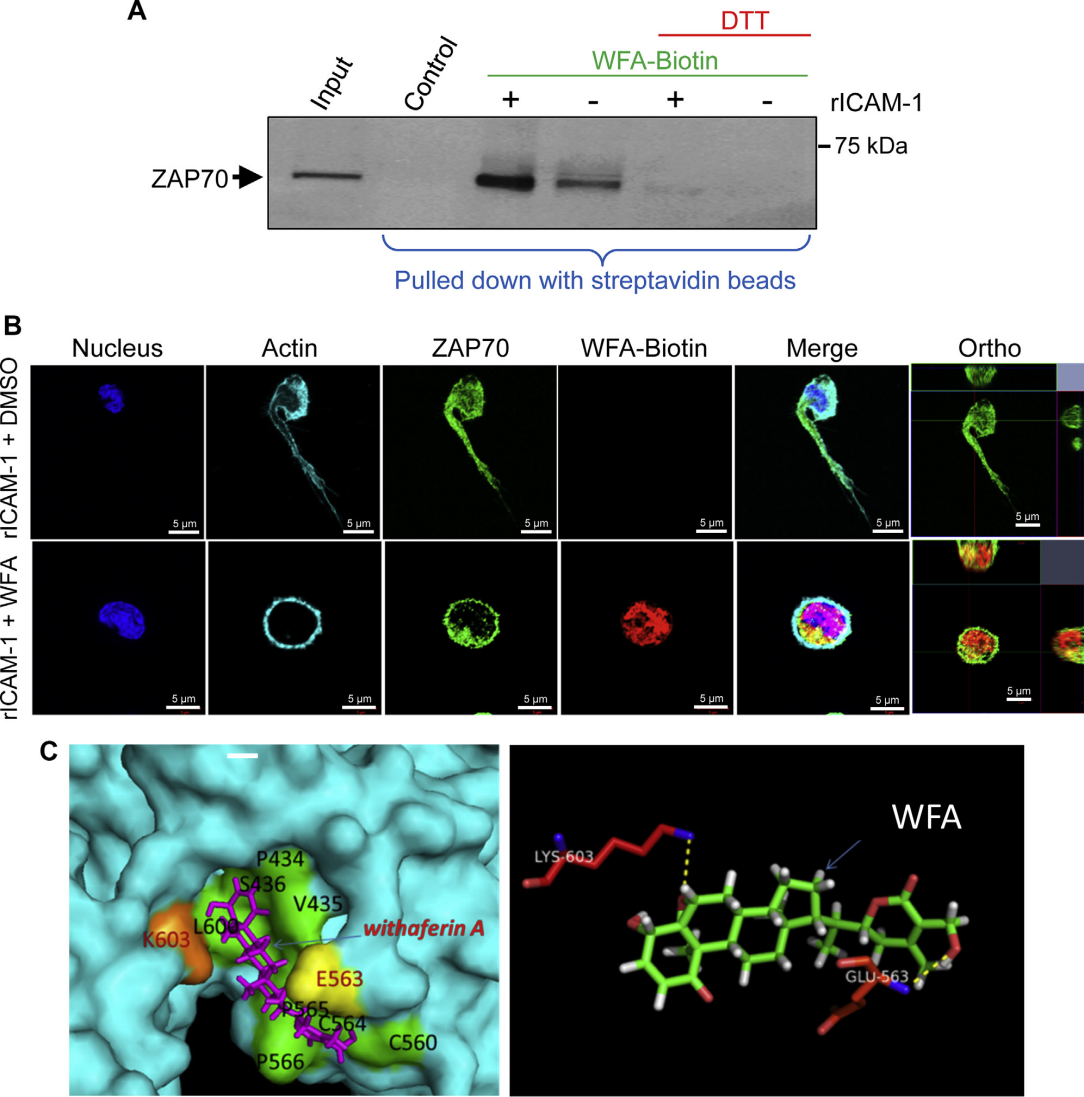
**WFA binding to ZAP70 in human T-cells.***A*, human primary T-cells pretreated with WFA-Biotin for 3 h or DMSO were stimulated to migrate on rICAM-1-coated plates for 30 min and lysed. Cellular lysates were immunoprecipitated with streptavidin-coated agarose beads and then Western immunoblotted for ZAP70. Cellular lysates from DTT-treated cells were evaluated for WFA-Biotin binding as control, whereas T-cell lysates not mixed with WFA-Biotin were used as an input control. *B*, LFA-1/ICAM-1-stimulated T-cells either treated with DMSO or WFA-Biotin were immunostained with anti-ZAP70/Alexa Fluor 488 (*green*), streptavidin/Alexa Fluor 555 (*red*), phalloidin-Alexa Fluor 647 (actin, *cyan*), and Hoechst (nuclei, *blue*) and then imaged by confocal microscopy. Data represent at least three independent experiments. Scale bar = 5 μM. *C*, *in silico* analysis of WFA-ZAP70 interaction indicating hydrogen bonding and hydrophobic interactions.

We validated the WFA-ZAP70 interaction, which was identified in the proteomics analysis, by a bait-pulldown approach of ZAP70-WFA-Biotin complex on streptavidin. Western immunoblot probing with anti-ZAP70 showed specific bands associated with ZAP70 in both resting and LFA-1/ICAM-1-stimulated T-cells (Fig. 6*A*). WFA participates in Michael addition thioalkylation reactions *via* its epoxide or lactone ring (17, 18, 19). The competitive thiol donor dithiothreitol (DTT) is known to interrupt such reactions by WFA to several protein kinases and phosphatases with an SH domain in their catalytic sites (17, 20). To determine whether DTT disrupts WFA binding with the ZAP70 protein, we treated cells with 2 μM DTT. Data showed that WFA-ZAP70 interaction was lost in the presence of DTT in both unstimulated resting and LFA-1/ICAM-1-stimulated migrating T-cells (Fig. 6*A*). The WFA-ZAP70 interaction was further verified by molecular imaging of WFA-biotin using Alexa Fluor 555 streptavidin (Fig. 6*B*). Molecular modeling and *in silico* docking predicted hydrogen bonding between WFA and amino acid residues K603 and E563 of ZAP70 and hydrophobic interactions with P434, V435, S436, C560, C564, P565, P566, and L600 (Fig. 6*C*). In contrast, *in silico* docking of a WFA analogue, withanone (WN), showed hydrogen bonding/hydrophobic interactions between several residues on ZAP70, distinct from residues in the complex with WFA (Fig. S3). Since DTT is known to reverse WFA-mediated suppression of kinase activity by blocking alkylation of thiol-sensitive redox pathways (15, 17, 20) and DTT treatment abrogated WFA-ZAP70 interaction in our bait-pulldown assay, the most probable thiol donors in the docking complex could be C560, C564, or both residues.

### WFA abrogates LFA-1/ICAM-1 stimulated phosphorylation of MLC-Ser19

**Figure 7:**
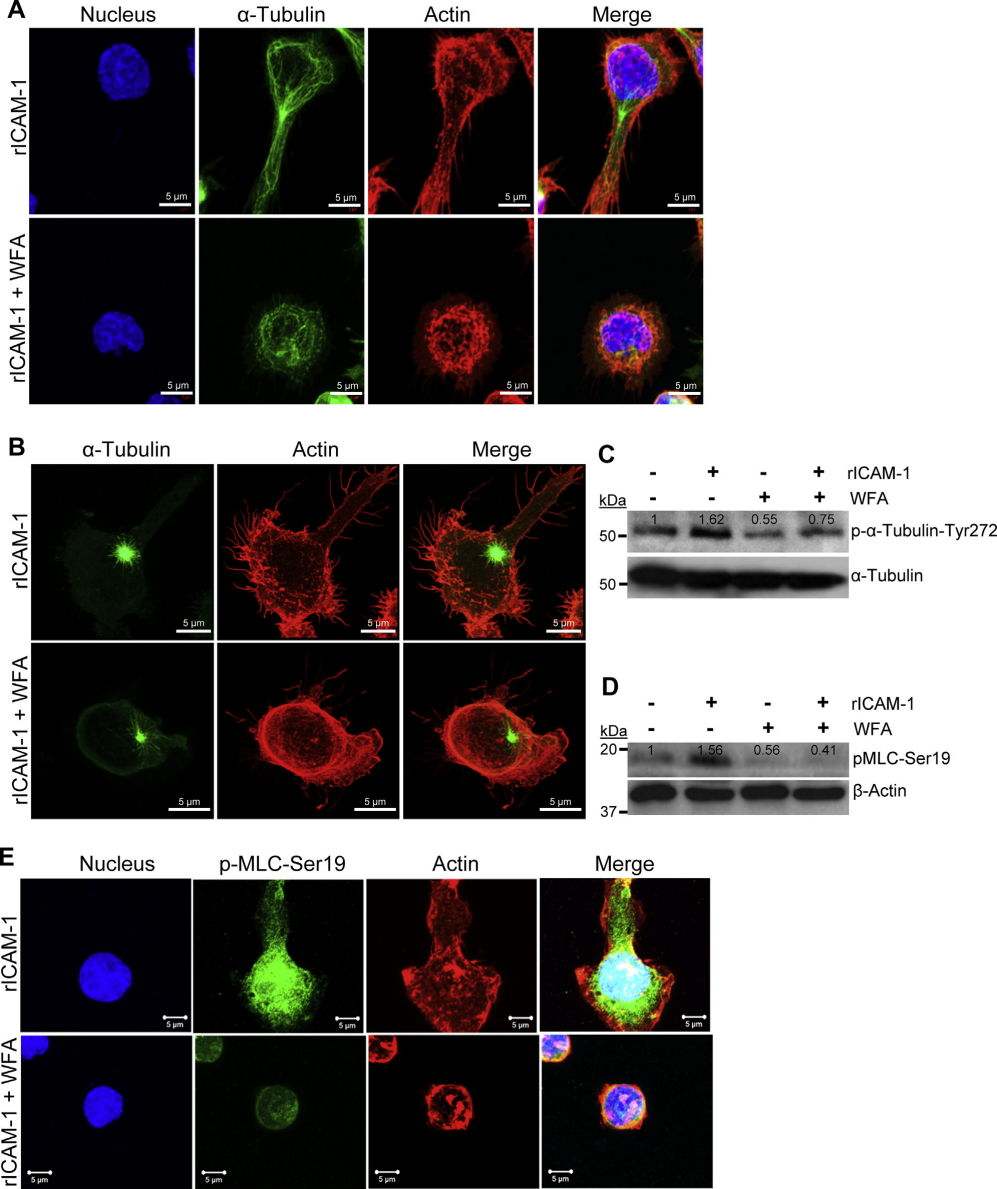
**WFA abrogates MLC phosphorylation in LFA-1/ICAM-1-stimulated motile T-cells.***A*, human primary T-cells either treated with 1.25 μM WFA or DMSO were stimulated to migrate on rICAM-1-coated surfaces for 30 min and fixed or lysed. Confocal microscopy images of T-cells stained with anti-α-tubulin-FITC (*green*), Alexa Fluor 568 Phalloidin (*red*), and Hoechst (nucleus, *blue*). Scale bar = 5 μM. *B*, control or WFA-treated T-cells seeded on rICAM-1-coated coverslips were subjected to microtubule depolymerization by incubating cells at 4 °C. Microtubule regrowth was analyzed by washing cells with prewarm medium and fixing after 15 s. AiryScan super-resolution microscopy was performed on cells stained with anti-α-tubulin-FITC (*green*) and Alexa Fluor 568 Phalloidin (*red*) to visualize tubulin regrowth and actin cytoskeleton, respectively. Scale bar = 5 μM. *C*, western immunoblots showing expression levels of p-α-tubulin-Tyr272 and α-tubulin (*loading control*) in unstimulated or LFA-1/ICAM-1-stimulated T-cells pretreated with 1.25 μM WFA for 3 h. *D*, western immunoblots showing expression levels of pMLC-Ser19 and β-actin (*loading control*) in unstimulated or LFA-1/ICAM-1-stimulated T-cells pretreated with 1.25 μM WFA for 3 h. *E*, confocal microscopy of untreated or WFA-treated LFA-1/ICAM-1-stimulated migrating T-cells stained with anti-pMLC-Ser19/Alexa Fluor 488 (*green*), Rhodamine Phalloidin 568 (actin, *red*), and Hoechst (nucleus, *blue*) indicating intense staining of pMLC-Ser19 at lamellipodial protrusions in migrating T-cells, abrogated by WFA. Scale bar = 5 μM. Data represent at least three independent experiments.

We next examined WFA regulation of T-cell cytoskeletal proteins—tubulin and actin. Although the characteristic migratory phenotypes were lost in WFA-treated LFA-1/ICAM-1-stimulated T-cells, confocal imaging indicated minimal changes in tubulin depolymerization and tubulin bundling architecture in WFA-treated T-cells (Fig. 7*A*). Consistently, the shift in tubulin detyrosination upon WFA treatment compared with untreated LFA-1/ICAM-1-stimulated migrating control T-cells was limited (Fig. S4*A*). Likewise, there was marginal disparity in nucleation from microtubule organizing center of acetylated tubulin in WFA-treated LFA-1/ICAM-1-stimulated T-cells compared with untreated control (Fig. S4*B*). Furthermore, in microtubule regrowth assays after 15 s of recovery, we observed the occurrence of microtubule nucleation centers in both WFA-treated and untreated LFA-1/ICAM-1-stimulated T-cells (Fig. 7*B*). These suggest that tubulin nucleation may not be a prime target for WFA-specific inhibition of LFA-1/ICAM-1-stimulated T-cell migration. Of note, a reduction in phosphorylated tubulin was observed using p-Tyr272 anti-tubulin antibody (Fig. 7*C*). Disrupted tubulin architecture could be observed upon prolonged incubation of WFA in LFA-1/ICAM-1-stimulated T-cells (Fig. S5). We argue that WFA could abrogate early-phase cytoskeletal dynamics by targeting actin-binding proteins, observed in the WFA-interactome in LFA-1/ICAM-1-induced migrating T-cells. Confocal imaging and Western immunoblotting further confirmed the activity of WFA in depleting pMLC-Ser19 levels in LFA-1/ICAM-1-stimulated T-cells (Fig. 7, *D* and *E*). Overall, these datasets indicate that WFA abolishes LFA-1/ICAM-1-induced phosphorylation of MLC-Ser19 in motile T-cells.

## Discussion

The findings presented herein propose a novel molecular mechanism of WFA action by specific regulation of LFA-1/ICAM-1-stimulated signaling in motile T-cells. T-cell motility is a multistep process requiring an array of signaling involving many kinases triggered *via* adhesive interactions between LFA-1 and ICAM-1, although a small fraction of T-cell blast subpopulation is inherently motile. By providing multiple lines of evidence, we demonstrate that WFA targets the ZAP70 kinase by direct binding and thus inhibits downstream signaling abrogating LFA-1/ICAM-1-stimulated T-cell migration. *First*, WFA did not interfere with the T-cell adhesion on rICAM-1. *Second*, WFA inhibited TCR-induced production of cytokines, independent of a direct LFA-1/ICAM-1 binding. *Third*, the majority of WFA colocalized with ZAP70, and not on the LFA-1-rich surfaces of motile T-cells. *Fourth*, pull-down of cellular proteins using WFA-Biotin/streptavidin beads and subsequent mass-spectrometry analysis identified ZAP70 as one of the WFA interacting proteins, and we did not detect LFA-1 in the WFA-interactome. *Fifth*, the main experimental variable used in the current study was no treatment or WFA treatment of T-cells followed by stimulation *via* LFA-1/ICAM-1 binding. The surfaces that were used to stimulate T-cells were fully covered with rICAM-1 coating. Only in the high content analysis assay, cells were incubated on poly-L-lysine coated surface for the purpose of capturing and imaging, as a control displaying a typical round shape of unstimulated resting T-cells. In our experimental model and assays, we treated T-cells with WFA before incubating them on surface immobilized rICAM-1, ruling out the possibility of WFA binding to ICAM-1. *Finally*, LFA-1 *outside-in* signaling (following LFA-1/ICAM-1 binding) failed to induce ZAP70 phosphorylation in WFA-treated T-cells. Migration assays on rICAM-1-coated plate contained MgCl_2_ and EGTA in the cell culture medium to induce the high affinity form of the LFA-1 receptor on T-cells. It is possible *in vivo* that the inhibition of ZAP70 by WFA would also impair *inside-out* signaling that would prevent or delay the expression/formation of high-affinity LFA-1 on T-cells and subsequent motile events, such as adhesion strengthening and migration necessary for T-lymphocyte responses when stimulated vasculature is encountered at sites of infection or injury.

Research over the past decade has established that WFA targets multiple cellular pathways (including the NF-κB and STAT3 pathways) and processes (such as angiogenesis and tumor invasion and metastasis). It has been shown that WFA improves functional recovery in several inflammation model systems, presumably by inhibition of NFκB pathway (21, 22, 23, 24). The identification of WFA-interactome associated *via* ZAP70, TCR, and NFκB in T-cells could be important in understanding T-cell dysfunctions acquired through prolonged exposure to TNF-α in autoimmune diseases. Further examination of WFA interactions with glucose-6-phosphate dehydrogenase, fatty acid synthase, and mTOR identified in the chemo-proteomic analysis will reveal WFA function in redox sensitive immunometabolism and nutrient signaling networks in T-cell migration and inflammation.

The WFA-specific phosphopeptidome analyses performed here revealed specific repression of various phospho-Tyr and phospho-Ser/Thr peptides, which is consistent with previous whole cell phosphoproteomics performed in activated T-cells (25). Previously, we and others have demonstrated the importance of ZAP70, PKC isoforms, AKT, TALIN1, and MAPK signaling pathways in LFA-1/ICAM-1-induced T-cell migration (26, 27, 28). The identification of SYK, MET, AKT, ERK2, PKCε, and FAK kinases in LFA-1/ICAM-1-stimulated T-cells in the current study emphasizes the importance of PI3K-complex signaling in T-cell extravasation and its inhibition by WFA upon treatment. In fact, various pharmacological inhibitors of PI3K are known to reduce inflammation and mitigation of PI3K signaling is extensively studied in attenuation of autoimmune diseases (29, 30).

The current research presents a comprehensive picture of WFA's activity by elaborating its inhibitory role in TCR-related kinase signaling (*e.g*., ZAP70, LAT, LCK, TXK). The role of ZAP70 activation through phosphorylation at various sites in T-cell migration and function has been previously described (9). The observed impact of WFA on LFA-1/ICAM-1-induced phosphorylation of ZAP70, LCK, LAT, and SLP76 suggests its crucial implication in TCR-kinase signals and T-cell effector functions. Moreover, we showed that WFA interaction with ZAP70 could be abrogated by the thiol donor DTT, predicting a favorable WFA binding to cysteines 560 and/or 564 as potential thiol donors in the inhibition of ZAP70 activity. Of note, a C564 mutation in the tyrosine kinase domain of ZAP70 was previously reported in severe combined immunodeficiencies (31). It should also be noted that DTT may impact T-cell surface proteins (32).

By employing ZAP70 modeling and WFA docking, based on the success of such exercises in the prediction of probable residues involved in molecular interactions (33, 34), we identified C560/564 as WFA binding sites on ZAP70. A combination of multiple methods, including experimental confirmation of adducts and solving structural complexes with analogues of WFA (35), would pinpoint the exact residues involved in WFA-ZAP70 interactions. Differential binding interactions of WFA and WN with amino acid residues on ZAP70 observed in the current docking simulations may be attributed to various factors, such as structural conformations, molecular size, and the position of the functional groups.

Translation of T-cell adhesion to cellular migration or function is achieved *via* mobilizing actin linkers (*e.g.*, Talin-1), protein kinase C (PKC) β1- and PKCε-dependent microtubule rearrangements and several protein–protein interactions that facilitate LFA-1 *outside-in* signaling (10, 36, 37). The potential of WFA to bind and modulate cytoskeletal elements through actin microfilament aggregation or tubulin depolymerization in other cellular model systems has been described (38, 39, 40).

Two populations of microtubules, dynamic (tyrosinated) and stable (detyrosinated), exist in T-cells and the tyrosinated microtubule fraction increases following stimulation (41). Moreover, an SYK-dependent phosphorylation of tubulin was reported in lymphocyte activation (42, 43). However, the effect of LFA-1/ICAM-1-stimulation that reduces stable microtubule fraction (by decreasing detyrosinated tubulin) remained relatively similar in WFA treated or untreated motile T-cells. Tubulin regrowth assays showed normal tubulin nucleation even in WFA-treated LFA-1/ICAM-1-stimulated T-cells. This may be due to the concentration and time of treatment employed in our experiments, which targeted early-phase dynamics in T-cell migration. Although total α-tubulin did not show any structural change in confocal imaging (namely tubulin bundling or depolymerization) in the context of early dynamics of T-cell migration, the effect of WFA on tubulin-cytoskeleton appears to be mitigating Tyr272 phosphorylation of α-tubulin.

Kinase signaling induces a coordinated polarization of actin cytoskeleton that is required for T-cell motility (44). The instantaneous intracellular changes arising from LFA-1/ICAM-1 ligation in T-lymphocytes in the early phase lead to ROCK or MLCK kinases-dependent acto-myosin contraction, to initiate dynamic reorganization of cytoskeleton (45). Posttranslational modification of MLC pivots the migratory apparatus at the leading edge of a migratory T-cell to facilitate cytoskeletal modulations (46, 47). Phosphoproteomics studies had previously described p-MLC-Ser19 as the primary site of phosphorylation in active T-cells (48). It can be argued that WFA-induced inhibition of p-MLC-Ser19 observed in the current study may not be dependent on WFA's inhibitory action on ZAP-70, but possibly through myosin light chain kinase or Rho Kinase. A crucial role of these two kinases in regulating myosin motor activities in LFA-1/ICAM-1-induced T-cell motility has been reported (45). Therefore, the inhibition of p-MLC-Ser19 by WFA in T-cells is another critical factor in the cessation of LFA-1/ICAM-1-stimulated T-cell migration.

The LFA-1/ZAP70 complex is known to recruit other proteins, such as Talin1, ARP-2/3, and WASP to the lamella of the polarized T-cell to facilitate actin reorganization (49, 50). Interestingly, proteins in the LFA-1/ZAP70 complex that help in actin reorganization were found to be modulated by WFA in LFA-1/ICAM-1-stimulated migrating T-cells in proteome. However, some of these proteins may well have been pulled down due to scaffolding activity of cytoskeletal elements, rather than immediate interaction with WFA. A comprehensive mapping of WFA activity in LFA-1/ICAM-1-stimulated T-cells suggested two modes of actin cytoskeletal regulation in early-phase migration. One is by inhibition of cortactin and ARP-2/3 complex and the other by regulating α-actinin (Fig. S6).

The integrin-dependent adhesion and subsequent migration of T-cells are regulated by both affinity and valency, which provide the avidity and further strengthen the interaction between LFA-1 and ICAM-1. Shear forces also induce ICAM-1 nanoclustering on endothelial cells that impact on T-cell motility (51). A better understanding of cellular signaling dynamics and adaptive mechanisms in T-cells is critical for successful therapeutic strategies against chronic inflammatory or autoimmune diseases. Apart from providing a catalogue of phosphorylation events during LFA-1/ICAM-1 engagement in T-cells, present study demonstrates novel kinases as targets of WFA involved in regulation of human T-cell migration *via* LFA-1/ICAM-1 engagement and thus has profound therapeutic implications for inflammatory conditions.

## Experimental procedures

### T-cell culture

Human peripheral blood T-lymphocyte blasts (referred to as primary T-cells in this article) were isolated from blood samples collected from healthy volunteers and blood packs obtained from the Health Services Authority of Singapore, as described previously (52). Briefly, peripheral blood mononuclear cells were isolated by density gradient centrifugation using the Lymphoprep density gradient medium and SepMate tubes (STEMCELL Technologies Inc). Monocytes were removed by plastic adherence at 37 °C. Suspension cells were cultured in Gibco RPMI-1640 containing 10% fetal bovine serum (FBS) and 2 μg/ml phytohemagglutinin for 3 days. After washing, cells were expended in RPMI-1640 containing 10% FBS and 20 ng/ml IL-2 and used for migration experiments between day 7 and day 14. This protocol results in >98% CD3^+^ T-cells and typically approximately 83% being CD4^+^ T-cells (53). Experiments were approved by the Institutional Review Board of Nanyang Technological University, Singapore (IRB-2014-09-007 and IRB-2018-05-034). The human T-cell line HuT78 (ATCC TIB-161TM) was cultured as described previously (54). Briefly, cells were cultured in RPMI-1640 containing 10% FBS and 2 mm L-glutamine. Gibco penicillin-streptomycin (1%) antibiotics (Thermo Fisher Scientific) were added to all the culture media. Cells were maintained in a humidified chamber at 37 °C containing 5% CO_2_.

### Induction of LFA-1/ICAM-1-stimulated T-cell migration

T-cells were stimulated to migrate *via* LFA-1/ICAM-1 adhesive interactions using our well characterized model system, as described (26, 55). Briefly, 96- or 6-well flat bottom Nunc tissue culture plates (Thermo Fisher Scientific) were coated with 5 μg/ml Fc-specific goat anti-human IgG in sterile phosphate buffered saline (PBS) overnight at 4 °C. After washing with PBS, human rICAM-1-Fc (1 μg/ml, Sino Biologicals) was added to the wells, and the plates were incubated further for 2 h at 37 °C to fully coat the well surfaces. T-cells, pretreated with 5 mM MgCl_2_/1.5 mM EGTA in the cell culture medium for 10 min to induce the high-affinity form of the LFA-1 receptor, were loaded into the rICAM-1-coated wells (2 × 10^4^ cells or 60 × 10^4^ cells per well in 96- or 6-well plates) and incubated in a cell culture incubator at 37 °C and 5% CO_2_ for specific time periods, as indicated in the figure legends of the particular experiments.

### High content analysis

A previously optimized high content analysis protocol for T-cell migration and phenotypic quantification was used (56). LFA-1/ICAM-1-stimulated T-cell migration was induced by incubating cells in the wells of 96-well plates that were fully coated with rICAM-1, as described above. T-cell resting-controls were added onto the poly L-lysine (PLL)-coated wells and cells pretreated with taxol were used as a control for migration inhibition. WFA (Chromadex) at various concentrations (0.3 μM to 2.5 μM) were used to treat T-cells prior to migratory stimulus *via* LFA-1/ICAM-1 binding. At the end of the experiments, cells were fixed with 3% (*v/v*) formaldehyde in PBS, fluorescently stained with Alexa Fluor phalloidin 647 or Rhodamine-Phalloidin (Life Technologies) and Hoechst 33258 (Sigma-Aldrich). Plates were then scanned (16 randomly selected fields/well at 20× objective) using the IN Cell Analyzer 2200 automated microscope (GE Healthcare). T-cell migratory phenotypes and cell adhesion on rICAM-1 were automatically quantified into calculated cell area, nuclear displacement, cell 1/form-factor, and cell numbers. The data were normalized and converted to box and violin plots for better visualization by GraphPad Prism (Version 8.0, GraphPad Software).

### Transwell migration assay

The assay for transwell migration of T-cells was performed using the RTCA CIM-Plate 16 (Agilent). Briefly, upper chambers of the electronically integrated CIM-Plate 16 were precoated with 5 μg/ml rICAM-1-Fc at 4 °C overnight and blocked with 5% (w/v) bovine serum albumin for 1 h at 37 °C. Control or WFA-treated serum starved T-cells in cell culture medium containing 5 mM MgCl_2_/1.5 mM EGTA were loaded in triplicate in the CIM-Plate 16 chambers (10 × 10^4^ cells/well). Cells were allowed to transmigrate toward 50 ng/ml SDF-1α-enriched serum-free medium in the lower wells at 37 °C for up to 16 h. Transwell migration of T-cells was monitored automatically in real time using impedance-based measurements by the xCELLigence system (Agilent).

### Measurement of T-cell adhesion on rICAM-1 under continuous shear flow

Adhesion of T-cells on rICAM-1 under continuous shear flow was determined as described earlier (57), with minor modifications. Briefly, biochips (Cellix Vena8 Endothelial^+^, Cellix Ltd) were coated with 5 μg/ml goat anti-human IgG (Fc specific) in sterile PBS for 2 h, at 37 °C. Following incubation, channels were washed with sterile PBS, followed by coating with 1 μg/ml rICAM-1-Fc at 37 °C for 2 h. The channels were then washed with PBS using Cellix Mirus Evo Nanopump (Cellix Ltd). Control or WFA-treated primary T-cells were introduced into different channels of the Biochip at a shear stress of 0.5 dyne/cm^2^ using Mirus Evo Nanopump. Adherence/migration of the T-cells was captured in a time-lapse sequence of one frame per second over a period of 5 min using Nikon Eclipse Ti Microscope (Nikon). T-cell adherence to rICAM-1 under flow was subsequently quantified by double blind counting of “in-focus” cells (flowing or nonadherent cells remain out of focus).

### Cell viability assays

Cell viability was determined using CellTiter 96 Aqueous One solution according to the manufacturer's instructions (Promega).

### Affinity purification and LC-MS/MS analysis

WFA-Biotin was synthesized as previously described (WFA-Biotin; University of Antwerp, Belgium) (16). For identification of endogenous WFA target proteins, both untreated resting and LFA-1/ICAM-1-stimulated migrating T-cells were incubated for 3 h with WFA-Biotin. Cells were lysed in 0.5 ml lysis buffer and incubated on streptavidin agarose beads (Thermo Fisher Scientific) overnight and then resolved by SDS-PAGE. Lysates of DMSO-treated T-cells were used as technical controls for mass spectrometry studies.

Tandem mass spectrometry was performed on the *in-gel* digested proteins with trypsin (Promega), as described earlier (58). Briefly, peptides were separated using PepMap C18 (3 μm, 100 Å, Thermo Fisher Scientific) and analyzed using a Dionex Ultimate 3000 RSLCnano system coupled to a Q Exactive tandem mass spectrometry (Thermo Fisher Scientific). Separation was performed using solvent A (0.1% formic acid) and solvent B (0.1% formic acid in 100% ACN) at flow rate of 300 nl/min with a 60 min gradient. A full MS scan (350–1600 m/z range) was acquired at a resolution of 70,000 and a maximum ion accumulation time of 100 ms. The automatic gain control (AGC) settings of the full MS scan and the MS2 scan were 5E6 and 2E5, respectively. An isolation width of 2 m/z was used for MS2. Single and unassigned charged ions were excluded from MS/MS. To identify peptides, peak lists were created using the Proteome Discoverer version 2.2 software (Thermo Fisher Scientific) and then searched against UniProt human protein sequence database (download on 18 Mar 2016, 23741427 residues; 70,225 sequences). Raw data files were processed and converted to Mascot generic files format, and MS/MS spectra were submitted for database searching against the Swiss-Prot Human database with Mascot (v2.4.1, Matrix Science, Ltd). Data generated by Mascot were validated using Scaffold (59) (version 4.5.1, Proteome Software Inc). Protein identifications were accepted if they assigned at least two unique peptides and with 99% probability. The basic search parameters were as follows: Trypsin digestion with a maximum of two missed cleavages permitted. Peptide modifications included in the search were fixed modification carbamidomethyl (C) and variable modifications oxidation (M), deamidation (NQ), and phosphorylation (STY). Mass tolerances were set to 10 ppm and 0.8 Da for a monoisotopic precursor and fragment ions, respectively. A peptide threshold of 99%, minimum two unique peptides, and false discovery rate ≤1.9% were used as cutoff values. Search results were then exported to Excel (Microsoft) for further processing and comparisons and also checked manually for accuracy. A protein network was generated using IPA (http://www.ingenuity.com/) by uploading a.csv file containing the list of proteins with statistical plausible interactions of WFA in LFA-1/ICAM-1-stimulated motile T-cells generated by Scaffold. The network was then improved using the “Build” tool in IPA (a web-based application that enables analysis, integration, and interpretation of proteomics and other “omics” datasets) to expand the network among the list of proteins identified in the LFA-1/ICAM-1-inuced T-cell migration specific WFA-interactome.

### Kinome profiling

T-cell kinome profiling was performed as described previously (60). Briefly, 1 μg T-cell protein lysate from each sample was utilized in the PTK and STK Kinase PamChip peptide microarrays (PamGene) according to the manufacturer's protocol in triplicate. Protein lysates of stimulated (*via* LFA-1/ICAM-1 cross-linking) and unstimulated resting or WFA-treated human primary T-cells were loaded to PamChip arrays containing 115 and 106 immobilized peptides that served as substrates for PTK and STK kinases, respectively. Later, intensities of peptide phosphorylation were monitored by adding a FITC-labeled anti-phospho-tyrosine antibody (PTK array) antibody or a blend of anti-phospho-serine/threonine antibodies in combination with an FITC-labeled secondary antibody. Images were acquired using the CCD camera in the PamStation 12 and signal quantification on phosphorylated peptides was performed using the Bionavigator software (PamGene International). Peptide intensities data were log2 transformed and differences in phosphorylation among samples were determined using a nonparametric *t* test. Upstream kinase analysis was performed using the PamApp to identify potential kinases that were hyperphosphorylated or hypophosphorylated compared with respective controls. Kinases were ranked according to their specificity and sensitivity scores (determined by the group and number of peptides phosphorylated). Kinase statistics to measure statistical changes among the experimental groups analyzed were generated to indicate activity of the identified kinase: a positive score (>0) reflects kinase hyperactivation, whereas a negative score refers to lower kinase activity in comparison to the control group. The intensity values of peptides showing statistical differences (*p*-values < 0.05) together with the log fold change were exported to GeneGo MetaCore (Clarivate analytics) for pathway analysis.

### Western immunoblotting

Standard immunoblotting procedures were employed as described previously (56). Equal amounts of T-cell lysates were resolved on SDS-PAGE gels, transferred onto PVDF membranes, incubated with diluted primary antibodies and later with horseradish peroxidise (HRP)-conjugated secondary antibodies to visualize immunoreactive bands by LumiGLO chemiluminescent detection system (Cell Signalling Technology). The images obtained by light sensitive film (Thermo Fisher Scientific) or by the ChemiDoc imaging system (Bio-Rad) were analyzed by Image J software. All primary antibodies were purchased from Cell Signaling Technology, Inc except for rabbit anti-MLC phospho-Ser19 (Sigma Aldrich), rabbit monoclonal anti-detyrosinated tubulin, and phospho-tubulin Tyr272 (Abcam). HRP-conjugated goat anti-mouse IgG was from Dako (Agilent).

### Confocal microscopy

Confocal imaging of T-cells was performed as described previously (61). Briefly, untreated or WFA-treated T-cells adhering on rICAM-1-coated glass coverslips were fixed (3% (v/v) formaldehyde in PBS) and permeabilized (0.3% Triton X-100 in PBS). Cells were fluorescently labeled with appropriate primary and secondary antibodies, as indicated in the corresponding figure legends. After washing, coverslips were mounted on glass slides using fluorescence mounting medium (Dako). Confocal imaging was carried out by a laser scanning microscope (Zeiss LSM800 AiryScan, Carl Zeiss, Inc) using a 63×/1.4 N.A. oil immersion objective lens. At least five different microscopic fields were analyzed for each sample using ZEN lite 2.1 imaging software (Carl Zeiss).

### Microtubule regrowth assay

Microtubule regrowth experiments were performed as described previously (61). Briefly, microtubule regrowth after tubulin depolymerization in cold shock was analyzed. WFA-treated or untreated LFA-1/ICAM-1-stimulated T-cells were washed and fixed after cold shock recovery at 37 °C for 15 s and processed for AiryScan confocal microscopy (Zeiss LSM800 AiryScan, Carl Zeiss).

### Cytokine analysis

Primary T-cells, pretreated with 1.25 μM WFA or equivalent amount of DMSO, were reactivated for 16 h on 24-well plates that were precoated with 1 μg/ml anti-CD3 and soluble 0.2 μg/ml anti-CD28. The cellular levels of IL-2 and IFN-γ mRNA were quantified by RT-qPCR. Secreted levels of IL-2 and IFN-γ were quantified using human Ready-SET-Go ELISA kits (Thermo Fisher Scientific) according to the prescribed protocols.

### WFA-ZAP70 docking

The chemical structure of WFA was retrieved in 2D MDL/SDF format from PubChem database (http://pubchem.ncbi.nlm.nih.gov) before energy minimization procedure in Discovery Studio 2.5 (Dassault Systems BIOVIA). The binding pockets on ZAP70 structure (PDB ID: 1U59) were identified using CASTp server (http://sts.bioe.uic.edu/castp/index.html). Docking was performed using GOLD software (62), as described previously (63). The GOLD score was opted to select the best docked conformations of ZAP-70 in the active site. GOLD parameter file was used to derive empirical constraints used in the fitness function (namely H-bond energies, torsion potentials, and hydrogen bond directionalities). One complex was selected based on the top GOLD fitness score.

### Statistical analysis

Ordinary one-way ANOVA (for comparison among multiple experimental groups) using GraphPad Prism 4.0 software. For kinome analysis, a nonparametric *t* test, two-group comparison, and biological replication comparison tests integrated in the statistical app within BioNavigator software (Pamgene NVA) were utilized. For all data analysis, *p* < 0.05 was deemed statistically significant.

## Data availability

The mass spectrometry proteomics data have been deposited to the ProteomeXchange Consortium (64) *via* the PRIDE (http://www.ebi.ac.uk/pride) (65, 66) partner repository with the dataset identifier PXD026508. All other data generated during this study are included in this submitted article and its supporting information.

## Supporting information

This article contains supporting information.

## Conflict of interest

The authors declare no conflicts of interest with the content of this article.
