# Supplementary material for: The steroidal lactone withaferin A impedes T-cell motility by inhibiting the kinase ZAP70 and subsequent kinome signaling
Source: J Biol Chem. 2021 Nov 3;297(6):101377. doi: 10.1016/j.jbc.2021.101377 (PMC8637146; doi:10.1016/j.jbc.2021.101377)
Supplement: Figures S1–S6 [file mmc4.pdf]

Supplementary Information

The steroidal lactone withaferin A impedes T-cell motility by inhibiting the kinase ZAP70 and subsequent kinome signaling

Mobashar Hussain Urf Turabe Fazil, Chandra Sekhar Chirumamilla, Claudina Perez-Novo, Brandon Han Siang Wong, Sunil Kumar, Siu Kwan Sze, Wim Vanden Berghe, and Navin Kumar Verma

Supplementary Table S1:

Protein IDs from Scaffold analysis. To identify peptides, peak lists were created using the Proteome Discoverer version 2.2 software (Thermo Fisher Scientific) and then searched against UniProt human protein sequence database (download on 18 Mar 2016, 23741427 residues; 70225 sequences). Raw data files were processed and converted to Mascot generic files format and MS/MS spectra were and submitted for database searching against the Swiss-Prot Human database with Mascot (v2.4.1, Matrix Science, Ltd.). Data generated by Mascot were validated using Scaffold (version 4.5.1, Proteome Software Inc., Portland, OR USA). Protein identifications were accepted if they assigned at least two unique peptides and with 99% probability. The basic search parameters were as follows - Trypsin digestion with a maximum of 2 missed cleavages permitted. Peptide modifications included in the search were fixed modification carbamidomethyl (C) and variable modifications oxidation (M), deamidation (NQ) and phosphorylation (STY). Mass tolerances were set to 10 ppm and 0.8 Da for a monoisotopic precursor and fragment ions, respectively. A peptide threshold of 99%, minimum 2 unique peptides, and FDR ≤ 1.9% were used as cut-off values. Search results were then exported to Excel (Microsoft) for further processing and comparisons and also checked manually for accuracy.

Supplementary Movies:

T-cell adhesion on ICAM-1 under continuous shear flow. Adhesion of T-cells on rICAM-1 under continuous shear flow was determined using microfluidic cell migration assay. Briefly, Biochips Cellix Vena8 Endothelial+™ biochips, Cellix Ltd. Ireland were coated with 5 µg/ml of goat anti-human IgG (Fc specific) in sterile PBS for 2 h, at 37°C. Following incubation, wells were washed with sterile PBS, followed by coating with 1 µg/ml rICAM-1-Fc at 37°C for 2 h. The channels were then washed with PBS using Cellix's Mirus Nanopump (Cellix Ltd.). Untreated (**Supplementary Movie 1**) or WFA-treated (**Supplementary Movie 2**) primary T-cells were introduced into different channels of the Biochip at a shear stress of 0.5 dyne/cm^2 using Cellix Mirus™ Evo Nanopump (Cellix Ltd. Ireland). Adherence and migration of the T-cells was captured in a time-lapse sequence of one frame per second over a period of 5 min using Nikon Ti eclipse microscope (Nikon, Singapore).

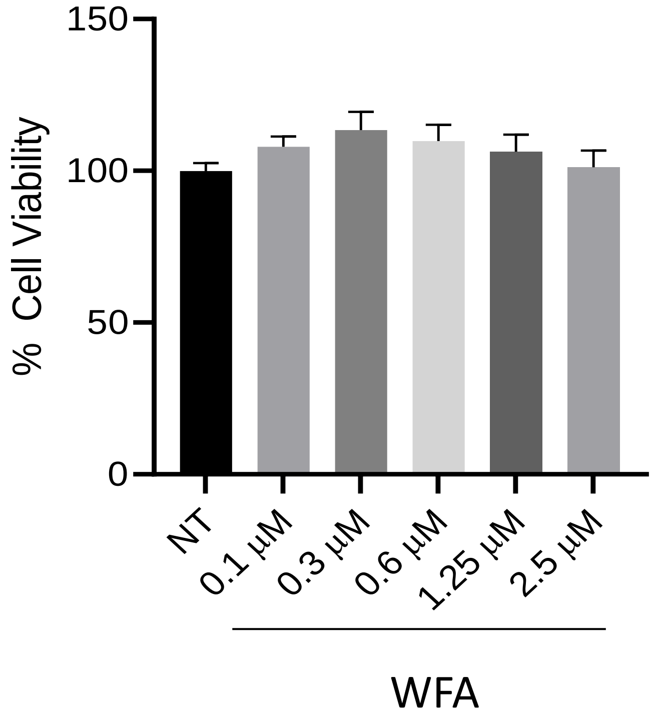

Figure S1. Effect of WFA on the viability of HuT78 T-cells. HuT78 cells (2×10^4 cells/per well in 96-well plates in triplicates) were treated with increasing concentrations of WFA for 3 h. CellTiter 96® Aqueous One Solution cell proliferation assay kit (Promega) was used to evaluate cell viability as per the manufacturer's instructions and absorbance was recorded at 490 nm. Absorbance readings were normalized against untreated controls and plotted as percentage (%) cell viability. Data shows mean ± SD of three independent experiments.

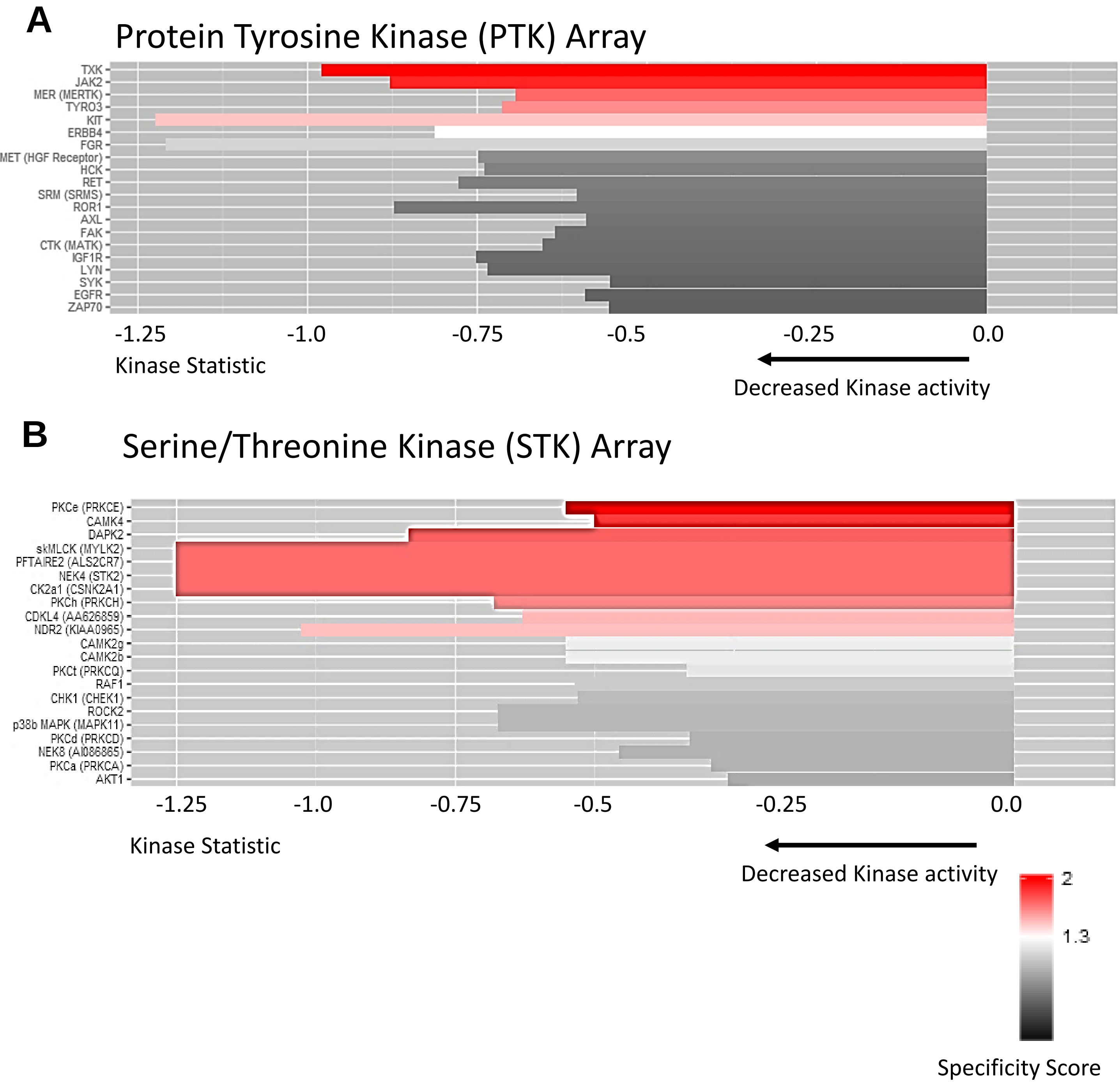

Figure S2. Upstream kinase analysis of WFA-treated human primary T-cells stimulated to migrate *via* LFA-1/ICAM-1 cross-linking. A normalized kinase statistic was calculated based on the significance of phosphorylation events and the specificity of the peptide sets that represent the corresponding kinase. The kinases ranked according to their specificity scores are shown in upstream kinase analysis of the PTK array (**A**) and STK array (**B**). The length of the bar indicates the measure of change in the WFA-treated LFA-1/ICAM-1-stimulated migrating T-cells compared to untreated migrating T-cells. The negative value indicates lower kinase activity in WFA-treated groups.

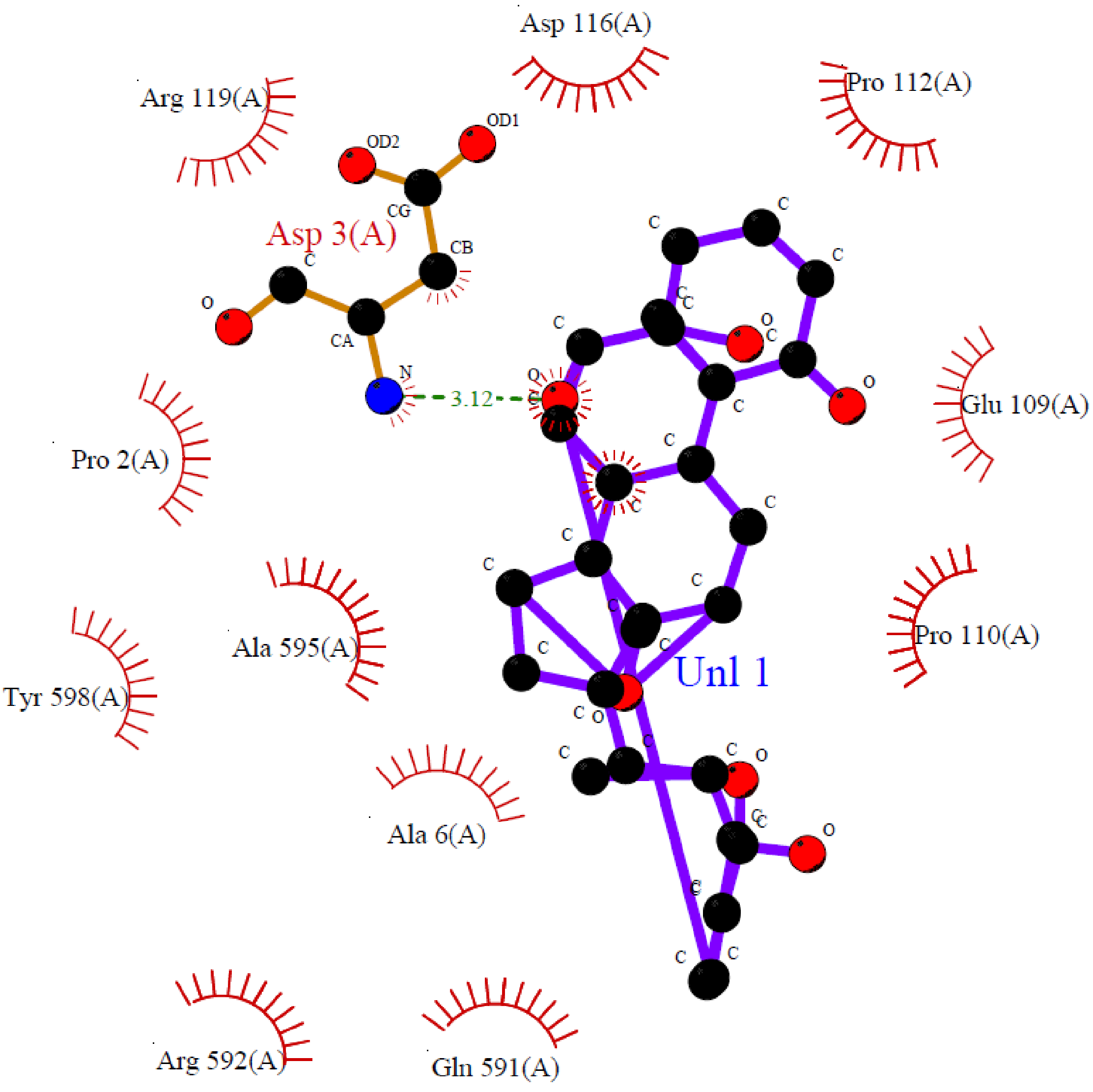

Figure S3. ***In silico*** **analysis of interactions between the ZAP70 protein and withanone (WN) indicating hydrogen bonding and hydrophobic interactions.**

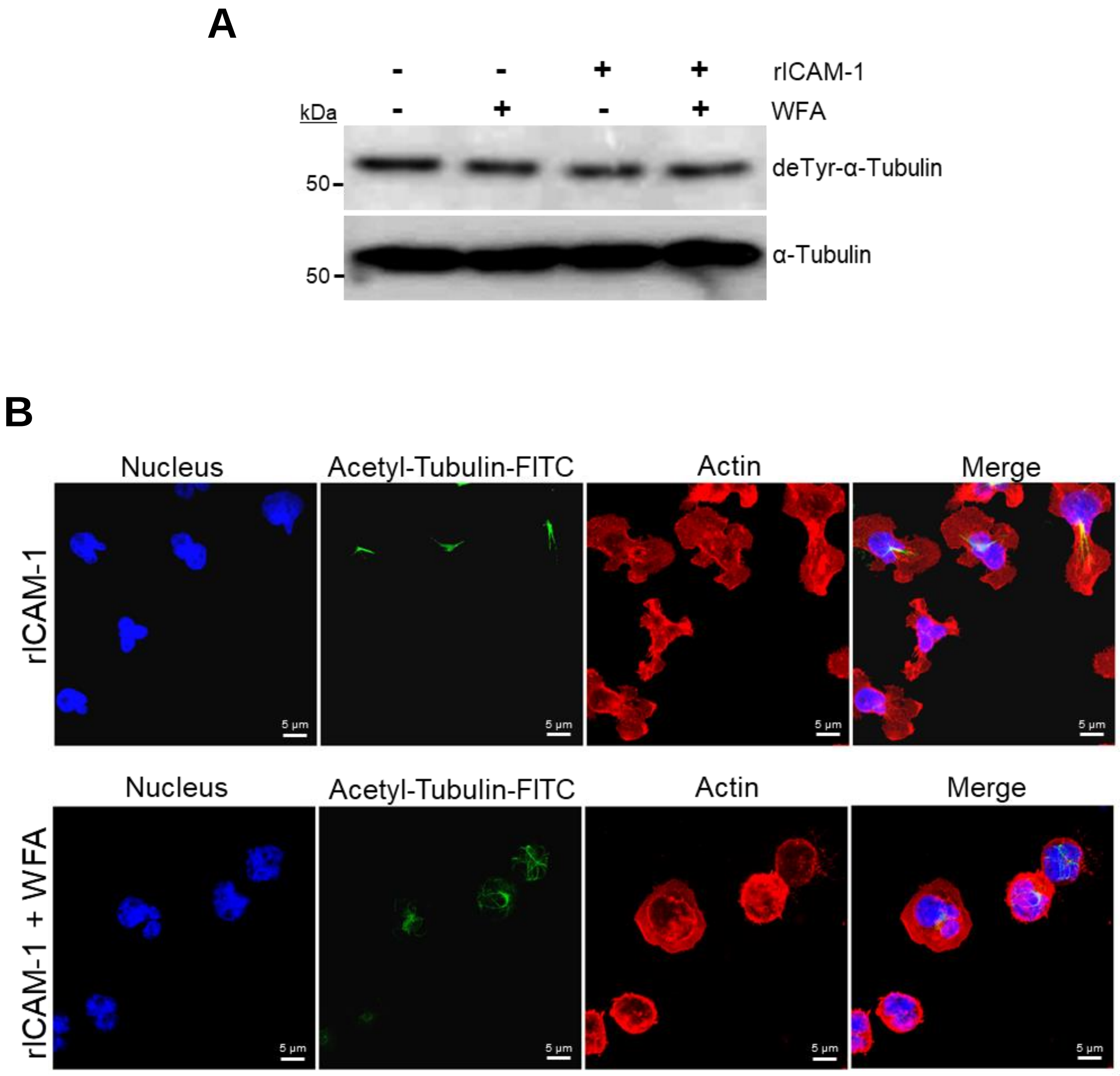

Figure S4. WFA-treated T-cells display analogous microtubule architecture compared to control. (**A**) Western immunoblots showing levels of detyrosinated α-tubulin, and total α-tubulin (l*oading control*) in unstimulated or LFA-1/ICAM-1-stimulated T-cells that were pre-treated with WFA. (**B**) Fluorescence microscopy of LFA-1/ICAM-1-stimulated T-cells that were pre-treated with WFA, stained for acetyl-tubulin-FITC indicating comparable zones of tubulin nucleation. Scale bar = 5 µm.

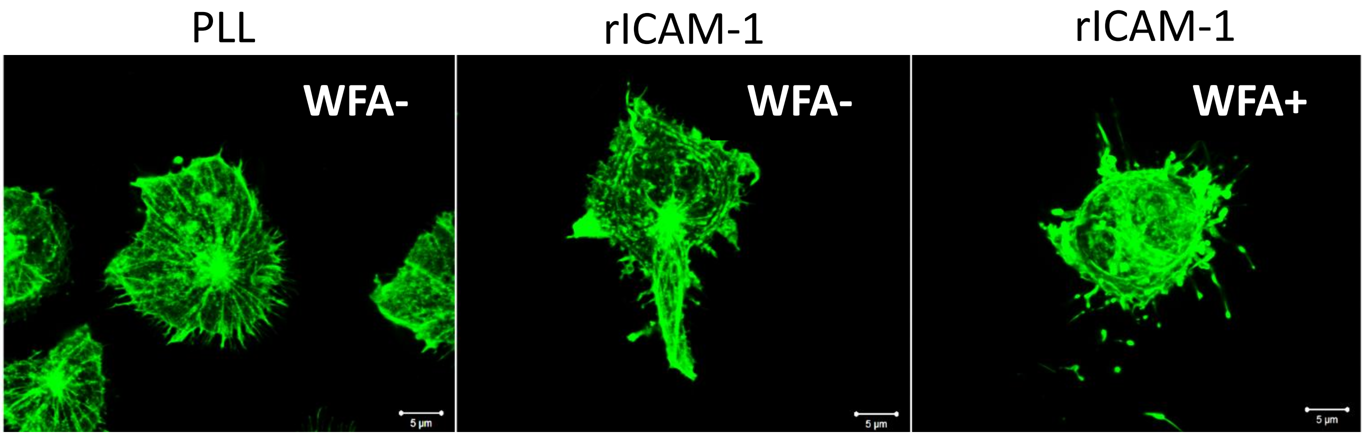

Figure S5. Long term WFA treatment disrupts microtubule architecture in LFA-1/ICAM-1-stimulated motile T-cells. AiryScan super-resolution imaging of tubulin in LFA-1/ICAM-1-stimulated T-cells indicating loss of characteristic microtubule structural illumination after 24 h WFA treatment. Scale bar = 5 µm.

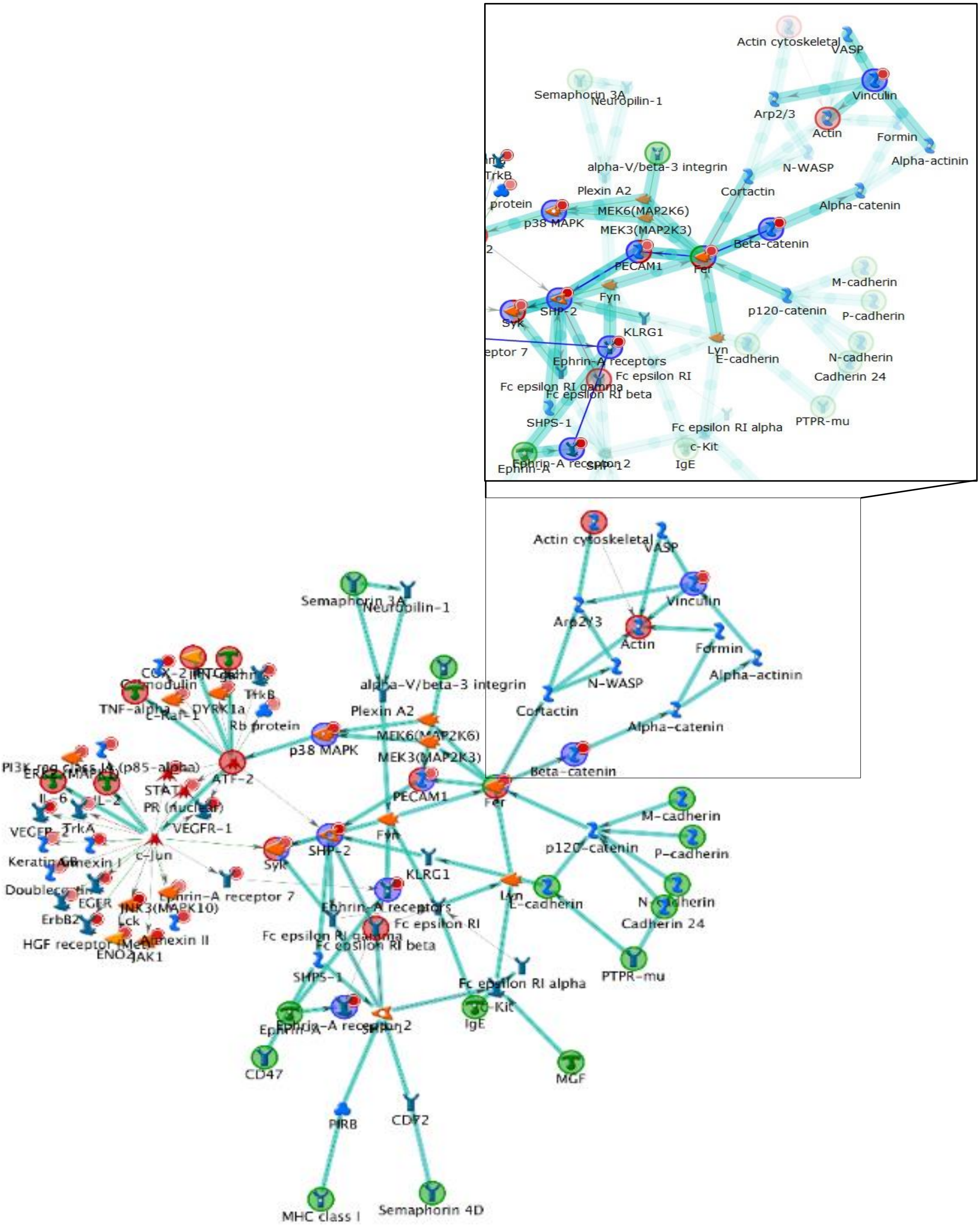

Figure S6. *GeneGo MetaCore®* Network Analysis of WFA interactome in LFA-1/ICAM-1-stimulated migrating T-cells demonstrates regulation of actin cytoskeleton. A comprehensive mapping of WFA kinome in LFA-1/ICAM-1-stimulated migrating T-cells map two modes of actin cytoskeleton regulation pivoted by p38 MAPK and SYK kinases through cortactin and alpha-actinin proteins.
